# Optical Microscopy and the Extracellular Matrix Structure: A Review

**DOI:** 10.3390/cells10071760

**Published:** 2021-07-12

**Authors:** Joshua J. A. Poole, Leila B. Mostaço-Guidolin

**Affiliations:** Department of Systems and Computer Engineering, Faculty of Engineering and Design, Carleton University 1125 Colonel By Drive, Ottawa, ON K1S 5B6, Canada; JoshuaPoole@cmail.carleton.ca

**Keywords:** ECM, microscopy, optics, imaging, collagen, elastin, fibronectin, proteoglycans, fibers, super resolution

## Abstract

Biological tissues are not uniquely composed of cells. A substantial part of their volume is extracellular space, which is primarily filled by an intricate network of macromolecules constituting the extracellular matrix (ECM). The ECM serves as the scaffolding for tissues and organs throughout the body, playing an essential role in their structural and functional integrity. Understanding the intimate interaction between the cells and their structural microenvironment is central to our understanding of the factors driving the formation of normal versus remodelled tissue, including the processes involved in chronic fibrotic diseases. The visualization of the ECM is a key factor to track such changes successfully. This review is focused on presenting several optical imaging microscopy modalities used to characterize different ECM components. In this review, we describe and provide examples of applications of a vast gamut of microscopy techniques, such as widefield fluorescence, total internal reflection fluorescence, laser scanning confocal microscopy, multipoint/slit confocal microscopy, two-photon excited fluorescence (TPEF), second and third harmonic generation (SHG, THG), coherent anti-Stokes Raman scattering (CARS), fluorescence lifetime imaging microscopy (FLIM), structured illumination microscopy (SIM), stimulated emission depletion microscopy (STED), ground-state depletion microscopy (GSD), and photoactivated localization microscopy (PALM/fPALM), as well as their main advantages, limitations.

## 1. Introduction

The extracellular matrix (ECM) is a three-dimensional structure of fibers, gels, and minerals (such as the hydroxyapatite found in bone [1]) that underlies all living tissues, forming the foundation on which cells sit. It provides mechanical support for the cellular component of tissues, regulation of chemical communication, and serves as a buffer for growth factors needed by cells [2]. The structure of the ECM has profound effects on the function of the tissue, beyond that which the cellular component of the tissue confers. For example, the ECM of blood is the plasma within which blood cells are suspended. Compared to the sheet-like deposition of collagen fibers found in basement membranes or the collection of collagen and minerals found in bone, different forms and structures of the ECM have vastly different effects on the tissue in which it is located. In opposition to the fiber-rich ECM of connective tissues, the ECM of the brain is fiber-scarce, rich in hyaluronic acid and proteoglycans, and specialized structures such as perineuronal nets [3,4,5]. The structure of the ECM is mainly conferred by the fibrous proteins found within themselves surrounded by gel-like proteoglycans [6,7,8].

However, despite the advances in several areas, little is known regarding the mechanisms by which specific structural and mechanical properties of the ECM influence its interaction with cells, especially within a tissue-like context or in tissue models, such as organoids and 3D-bioprinted models. This lack of knowledge prevents the understanding of cellular microenvironments regarding effective tissue repair, remodelling, and even the action of therapies. The visualization of key ECM components has been the subject of studies for several years. Every time a new imaging technology emerges, several applications focused on the characterization of cellular responses and the distribution, morphology, and changes within the ECM closely follow. In this review, we present an overview of the main components of the ECM and an extensive description of several optical microscopy techniques that have been used to track changes in the cellular microenvironment.

### 1.1. ECM Fibrous Proteins: Collagens, Elastins, Fibronectins, and Laminins

**Figure 1:**
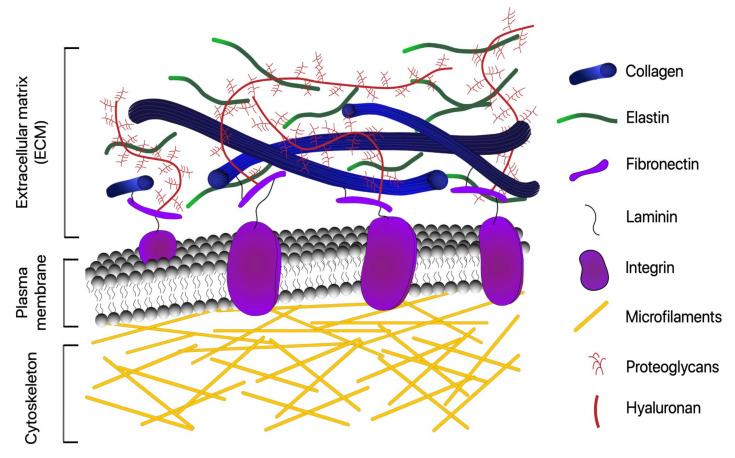
Schematic overview of extracellular matrix and its major components. Although the ECM composition varies depending on the tissue, the matrix is mainly composed of a variety of fibrous proteins (collagen, elastin, fibronectin, and laminin) and polysaccharides that are secreted locally and assembled into an organized meshwork in close association with the surface of the cell that produced them.

The fibrous proteins of the ECM consist of collagens [6,8,9,10], elastin [6,8,9,11], fibronectins [6,8,9,12], and laminins [6,8,9,13], as illustrated in Figure 1. Collagens make up a large portion of the total protein composition of the human body [8]. The common structure of all types of collagens is the triple helix [8]. Fibrillar collagens provide tissues with the structure for the attachment, regulation, proliferation, and migration of cells [8], and many of the mechanical properties of tissues, such as the strength of bone and tendon, or the ability to resist pressure in blood vessels, are inferred from the arrangement of fibrillar collagens [14].

Elastin is a unique protein that forms the bulk of elastic fibers, which impart elasticity and resilience to tissues [8]. Much like fibrillar collagen, the mechanical properties conferred on tissues by elastic fibers depend on how fibers are structured and distributed within a tissue [15].

Fibronectin (FN), much like elastin, is a unique, dimerized protein that forms fibrillar networks within the ECM [8]. The FN network has many different roles, such as cell adhesion, migration, proliferation, and differentiation [16], and binds many of the growth factors and signalling molecules needed by cells [12]. FN also has many implications in wound healing [17] and cancer vascularization and progression [8].

Laminins are noncollagenous proteins found as a major component of basement membranes, a specialized form of ECM that anchors cells and binds tissues [8]. Generally, the long chain of laminin interacts with cells via membrane receptors such as integrins, and the short chains interact with other extracellular proteins, such as collagen IV [8]. Laminins have a wide range of roles, from early embryonic development to angiogenesis [8].

### 1.2. ECM Proteoglycans and Hyaluronan

While collagens, elastins, and other fibrous proteins give structure and mechanical properties to the ECM, the space between them is filled with proteoglycans. Proteoglycans are composed of glycosaminoglycan (GAG) chains attached to a protein core [6]. They are highly hydrophilic, and due to their long, strand-like conformation, they form hydrogels that are crucial for the formation of matrices able to withstand high compressive forces [6].

As with the fibrous proteins of the ECM, different types and proportions of proteoglycans can impact the form and function of the tissue in which it is found. For example, versican is a proteoglycan found predominantly in the brain and blood vessels and has implications in tissue formation, cancer, and inflammation [18], or aggrecan, a proteoglycan found in load-bearing joints that has great resistance to compression [19,20].

**Figure 2:**
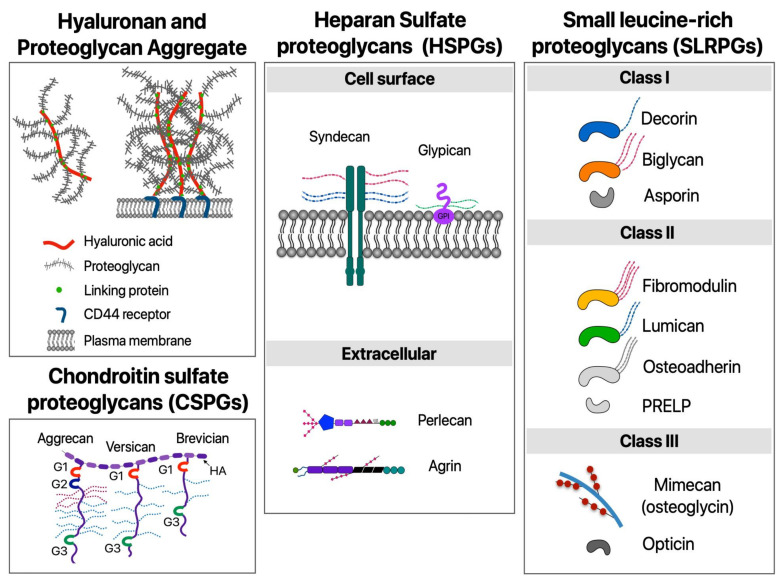
Simplified schematic of main proteoglycans found in the ECM. G1, G2, G3: Globular domains. HA: Hyaluronic acid. PRELP: proline/arginine-rich end leucine-rich repeat proteins.

Proteoglycans can be categorized into four main families: small leucine-rich proteoglycans (SLRPs), hylectans, pericellular and basement membrane proteoglycans, and cell-surface proteoglycans [7]. A schematic diagram of all families is shown in Figure 2. Cell-surface proteoglycans have a wide range of roles, from binding growth factors, cancer growth and development, and modulation of cell-surface topologies [7].

The last major extracellular glycosaminoglycan found in sizable amounts within the ECM is hyaluronic acid (HA) or hyaluronan. HA is found in many different tissues and has many roles [8], ranging from mechanical to chemical. Its hygroscopic characteristics hydrate the ECM, helping to regulate homeostasis and providing resistance against compression [21]. HA also acts as a lubricant in the synovial fluid of joints and binds to cell-surface receptors, acting as a signaling molecule [21].

### 1.3. The Role of the ECM in Tissue Repair and Chronic Diseases

While different configurations and proportions of the macromolecules of the ECM can drastically change the form and function of a tissue, changes in the state of the ECM also have implications for the health of the tissue. As tissue ages, it stiffens, a process mediated by the incorrect crosslinking of collagen fibers [22]. While the collagen network is modified, elastin and GAG levels are reduced [23,24], and tissue becomes stiffer, less elastic, and weaker as a result. While aging is a normal process all tissues go through, injury is one incident that occurs somewhat infrequently. Upon injury, wound repair mechanisms are activated at the site of injury, creating a cascade of chemical signals, originating with the detection of ECM-degradation products and ending with the proliferation and migration of fibroblasts through the damaged ECM [25]. Fibroblasts that have been activated and recruited in the repair of the ECM synthesize the macroproteins of the ECM and may differentiate into myofibroblasts, able to exert mechanical force on collagen fibrils of the ECM [26]. As novel collagen is deposited, crosslinking of the collagen bundles stiffens the tissue and provides directionality for cell migration into the damaged region [27,28]. After the wound has been repaired, restoration of homeostasis in the ECM is undertaken by feedback mechanisms to bring correct functioning back and return the tissue back from a fibrotic state [25].

However, should the injury become chronic or feedback mechanisms fail, the ECM will continue to be changed. The continuous remodelling of the ECM eventually alters the properties of the tissue and can lead to scar formation [29]. Therefore, characterization of how changes in the ECM are occurring can grant great insight into the state of the tissue, providing a window into how the ECM is behaving or changing over time. ECM remodelling can occur in all tissues, including lungs, liver, heart, and brain, and is a hallmark of several diseases, including certain types of cancer, atherosclerosis, and asthma [30,31,32,33,34,35,36].

Recent advances in the imaging of ECM components using different microscopy techniques have proven useful in enhancing our understanding of the supramolecular changes that occur during scar formation and disease progression. In addition, the visualization of key ECM proteins is crucial to reveal how changes in the cell microenvironment promote cellular responses leading to differentiation. In the next section, we discuss a set of optical imaging techniques for visualization of biochemically specific tissue features, including modalities that allow the visualization of ECM components such as collagen and elastin without the need for staining or sample preparation [37,38,39,40].

## 2. Imaging the ECM Components: Past, Present, and Future Challenges

The need to integrate new tools to better investigate the underlying mechanisms of diseases and characterize the various stages of tissue remodelling has received researchers’ attention for several decades. Optical microscopy has been a valuable tool for studying key facets of ECM remodelling. Some modalities and their label-free characteristics, combined with high sensitivity and specificity for major extracellular molecules, make them an attractive alternative (and a powerful ally) to conventional histology in studying tissue structures and ECM composition [37,41].

For example, fluorescence as an optical effect has been studied since the late 1800s, when visible light was observed emanating from objects that did not appear to reflect or have any apparent source of light [42]. While it was unknown how this effect was caused, further expansion into the physics of the matter found that light was absorbed by the substance and re-radiated in specific spectra [43]. Since the fluorophore absorbs one photon and one is emitted, these came to be known as linear optical processes. While this was very useful in categorizing inorganic substances, organic substances were also found to fluoresce, especially certain dyes and stains. Eventually, this led to the creation of specific fluorescent probes, known as fluorophores, that would bind to specific substances in cells and other biological material [44].

With specific probes for a plethora of compounds, chemicals, proteins, and processes found within living cells, fluorescence microscopy became a powerful tool for cell and molecular biologists [45] and remains a viable imaging technique for imaging and characterization of the ECM. Linear-fluorescence-based techniques can be broadly separated into two categories: (i) diffraction-limited microscopy and (ii) super-resolution microscopy, and the mainly used ones to image ECM components are mentioned in Table 1.

Classic light microscopy is limited in what resolution it can resolve by diffraction. This limit is defined by a point spread function (PSF), which is associated with the optical properties of the microscope. The PSF is the 3D diffraction pattern of light emitted from a very small point in the sample and transmitted to the image plane through the microscope objective. The PSF scales in size directly with the wavelength of light used and inversely to the numerical aperture of the microscope [46]. Thus, any object smaller than this fixed distance appears to be the same size as the PSF when observed. This limit has typically been ~250 nm in the X and Y direction and greater than 450–700 nm in the Z direction [46]. These diffraction-limited fluorescent techniques can be broadly categorized into four main groups: widefield fluorescence microscopy (WFFM), total internal reflection fluorescence microscopy (TIRFM), laser scanning confocal microscopy (LSCM), and slit confocal/spinning disk confocal microscopy (SC/SDCM), and their main advantages and limitations are summarized in Table 1.

While the resolution of classic light microscopy has been limited by diffraction to objects greater than a few hundred nanometers, obscuring the details of most cellular and acellular processes, several methods have been developed that circumnavigate this restriction in resolution, placed under the term ‘super-resolution microscopy’. Super-resolution techniques have allowed the imaging of fine details of the ECM, such as collagen fibrils, down to single-molecule imaging [47]. They can be broadly categorized into six groups: fluctuation-based, pixel reassignment, structured illumination microscopy (SIM), stimulated emission depletion microscopy (STED)/ground state depletion microscopy (GSD), single-molecule localization, such as photoactivated localization microscopy (PALM) and stochastic optical reconstruction microscopy (STORM), and expansion microscopy [47]. SIM and STED are commonly used in imaging ensembles or structures found within tissues, and PALM and STORM are best suited for single-molecule imaging [46]. The most common modalities, their advantages, and their main limitations are presented in Table 2.

**Figure 3:**
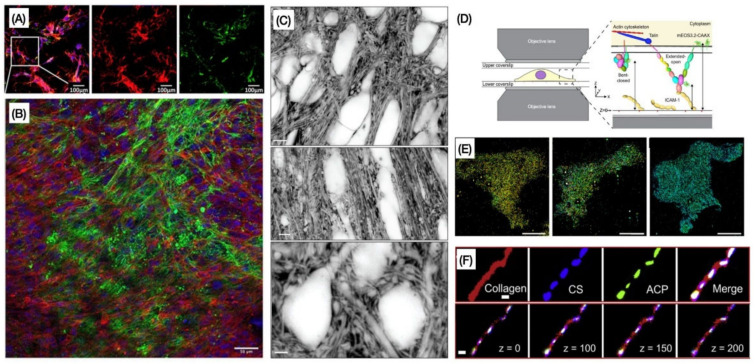
Representative images of different ECM components acquired by (**A**) WFFM, (**B**) LSCM, (**C**) STED/GSD, (**D**,**E**) PALM, and (**F**) STORM. (**A**) Shows a widefield fluorescence image of fibroblasts and matrix followed by images of Alexa-647-labeled fibronectin and antibody-stained collagen I alone. Scale bar, 100 μm [48]. (**B**) Image obtained by confocal microscopy of fibronectin deposition and localization in 3D co-cultures of human breast cancer cells and human dermal fibroblasts. Cultures were labelled to visualize actin (red), fibronectin (green), and cell nuclei (blue). Scale bar, 50 μm [49]. (**C**) Higher magnification SUSHI (super-resolution shadow imaging) images of cell bodies and neuropil in CA1 area. Scale bar in top, 4 mm; middle, 5 mm; bottom, 2 mm [50]. (**D**) Schematic of sample setup for iPALM imaging of migratory Jurkat T-lymphocytes adhered to ICAM-1 or fibronectin-coated lower coverslips, with gold nanorod fiducial markers (orange spheres) [51]. (**E**) Representative iPALM renderings of Jurkat cells expressing mEOS3.2-LFA-1 fusion, mEOS3.2-CAAX, or LifeAct-mEos3.2. Scale bars, 5 mm [52]. (**F**) STORM images of mineralized collagen fibrils at an early stage (top). Representative Z-dimension slices of the STORM images (bottom). Amorphous calcium phosphate (ACP) preferentially aggregates around purple in the intrafibrillar compartments, which represent chondroitin sulfate (CS) in CS-collagen fibril. Scale bar: 500 nm [52]. Images reproduced with permission from [48,49,50,51,52].

Combining different immunohistochemical stainings with specific microscopy modalities, various ECM components, and cells associated with it can be successfully visualized, including their 3D structure. To illustrate the capability of different modalities in imaging different ECM components, in Figure 3A, we show representative images of fibroblasts and the ECM secreted (fibronectin and collagen) imaged with WFFM [48]. In Figure 3B, LSCM is able to provide us with details of fibronectin deposition and localization in 3D co-cultures of human breast cancer cells and human dermal fibroblasts [49]. Super-resolution shadow imaging (SUSHI, shown in Figure 3C) is an excellent example of microscopy application in neuroscience, where images of cell bodies and neuropil can be seen with great detail [50]. iPALM imaging allows us to visualize, for example, migratory Jurkat T-lymphocytes adhered to ICAM-1 or fibronectin-coated lower coverslips, as shown in Figure 3D,E [51]. Finally, one example of STORM images is shown in Figure 3F, illustrating mineralized collagen fibrils as well as representative Z-dimension slices of the STORM images [52].

Linear optical methods rely on the absorption of single excitation light photons and the emission of single emission light photons; nonlinear methods, however, do not. The development boom in laser technology, which happened in the 1990s, allowed us to start exploring and applying the principles of nonlinear interactions between light and biological tissue [53,54]. In this context, the development of multiphoton excitation and multi-harmonic generation imaging represented the future direction in optical microscopy and the potential applications in biomedical science.

Other imaging modalities, such as electron microscopy or scanning probe microscopy, provided higher spatial resolution. However, optical microscopy modalities offered unique advantages to investigate biological structures, including the ECM. Nonlinear optical processes are harnessed in the following modalities: two-photon excitation (TPEF), second-harmonic generation (SHG), triple-harmonic generation (THG), and coherent anti-Stokes Raman scattering (CARS).

These modalities are often referred to as multiphoton microscopy in biomedical sciences, and some of their main advantages and limitations are summarized in Table 3.

**Figure 4:**
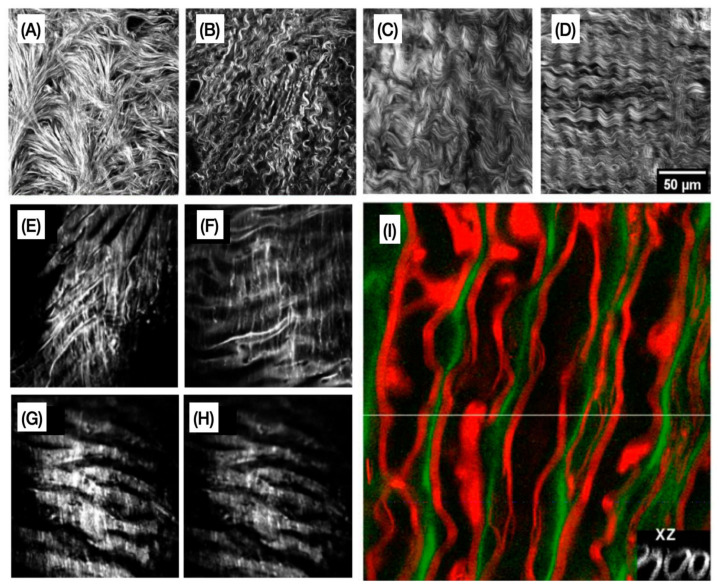
Representative multiphoton microscopy images. (**A**–**D**) Second-harmonic generation (SHG) optical sections of collagen from the four categories of ovarian tissues [55]. (**E**–**H**) Examples of two-photon emission fluorescence (TPEF) images of elastic fibers acquired from the regions along the aorta of myocardial infarction-prone Watanabe heritable hyperlipidemic rabbits [56]. (**I**) Simultaneous coherent anti-Stokes Raman scattering (CARS) imaging of axonal myelin and TPEF imaging of Oregon green 488 is represented by red and green colors. The grayscale inset image is an XZ image showing the cross-section of axons [57]. Images reproduced with permission from [55,56,57].

In Figure 4, we have examples of representative images obtained with SHG, TPEF, and CARS microscopy, illustrating the capability to assess ECM remodelling label-free and with high spatial resolution.

In the next sections, we discuss the various optical microscopy modalities and highlight their use in characterizing proteins of the extracellular matrix. Starting with linear optical methods, we present their use in imaging both intrinsic fluorophores of the ECM, such as elastin, and extrinsic fluorophores, such as Hoechst. Super-resolution techniques are also discussed as well as their use in imaging proteins found in association with the ECM, such as integrins. Nonlinear modalities are among the most powerful in imaging structural proteins found within the ECM, such as collagen and elastin. Finally, Raman-based modalities and their use in imaging some more exotic intrinsic fluorophores are also described. A summary of some representative works focused on imaging ECM components using different optical microscopy modalities is shown in Table 4.

### 2.1. Absorption, Scattering, Refraction, and Fluorescence: An Overview of Conventional (Linear) Optical Microscopy Modalities

**Figure 5:**
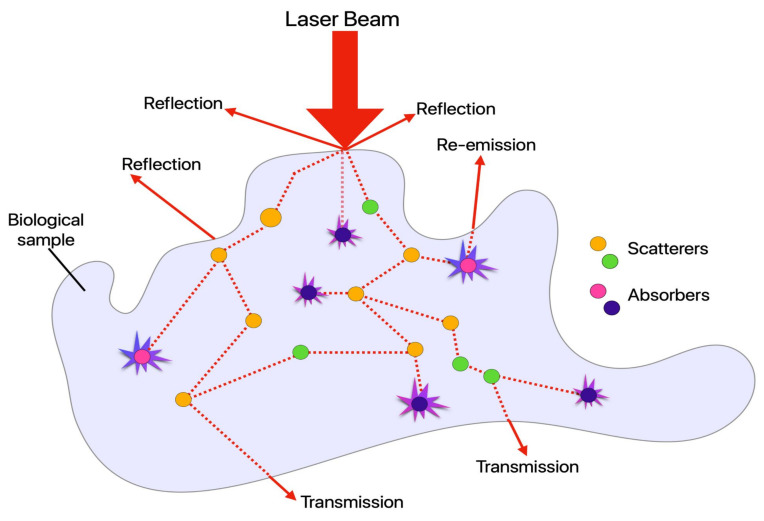
Primary effects of light–tissue interactions. Different components might act as scatterers or absorbers, depending on the wavelength of the incident light. Scatterers generate Raman signal, for example, while absorbers are endogenous or exogenous fluorophores as well as chromophores. Depending on the thickness of the sample (and the energy of the incident light), it might be possible to detect signal transmitted through the tissue.

Light interaction with biological samples (cells, tissues, or scaffolds) is complex. The different composition, layers, and optically inhomogeneous properties of biological samples allow light to be reflected at a material interface, refract when light enters a tissue structure that has a different refractive index, have the photon energy absorbed by the material, or have photons scattered in the material, as shown in Figure 5.

Even simply considering the ECM, the different fibrous proteins and components have different optical properties. However, in biological tissues, the variation in the refractive index is often small, and the tissues are, in many cases, transparent. One of the most significant interactions is the absorption of light. It determines how far it can penetrate a specific tissue and depends strongly on wavelength. The different absorption coefficients dictate how much energy a specific tissue can absorb from a particular optical source. Once the light is absorbed, it can be converted into heat, be radiated (fluorescence), or be consumed in photochemical reactions.

On the other hand, scattering is a phenomenon experienced by light as it travels through an inhomogeneous medium, such as biological tissues. While photons travel in a straight line from their source, small irregularities in the media through which they travel force them to deviate from this path. Light is scattered when induced dipoles within the media are inhomogeneous, deviating the photon from its path and producing different types of scattering, dependent on the wavelength of the incident light and the size of the scatterer [99].

While many types of scattering exist, the three most often seen are Rayleigh, Mie, and Raman [100]. Rayleigh scattering occurs when the wavelength of light is greater than the size of the scattering particle, and as it is an inelastic process, it scatters photons of the same wavelength as the incident light [100]. Mie scattering occurs when the wavelength of light is less than the size of the scattering particle. Raman scattering occurs due to inelastic collision between light and the scattering molecule. This can lead to three situations. Scattered light can have the same frequency (Raman scattering), scattered light can have a lower frequency (Stokes Raman scattering), or a higher frequency (anti-Stokes Raman scattering) [100]. In opaque tissues, such as the skin, light is highly scattered, whereas, in transparent tissues, such as the cornea, light is only weakly scattered [101]. Raman scattering is harnessed in certain imaging modalities, such as CARS.

While scattering changes the vibrational state of a molecule upon absorption of the incident light, fluorescence changes the electronic levels of a molecule [102]. Upon absorption of light, the molecule is excited to a higher electronic state [102]. This state eventually relaxes back to a ground state and, upon doing so, releases a single photon [102]. The effect of a single photon being absorbed and a single photon emitted is known as one-photon fluorescence [102]. Another significant difference between scattering and fluorescence is the time in which excited states exist. Scattering produces a short-lived, vibrational state, whereas fluorescence is a long-lived electronic state [102].

**Figure 6:**
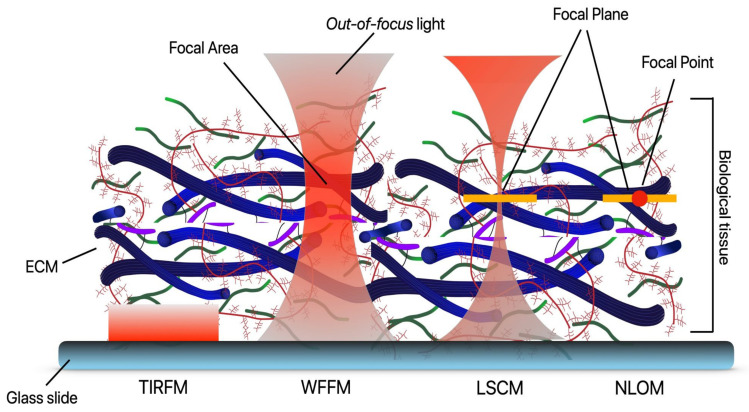
Schematic comparison of the most common optical microscopy modalities. TIRFM presents good SNR due to the low penetration depth of the evanescent field. WFFM is very versatile but tends to present a significant background signal. LSCM provides an improvement over WFFM by reducing the background detection and keeping its versatility. NLOM modalities only generate a significant signal on a specific focal point and have spatial resolution inferior to the so-called super-resolution modalities (e.g., SIM, STED, STORM, PALM). TIRF: total internal reflection fluorescence microscopy; WFFM: widefield fluorescence microscopy; LSCM: laser scanning confocal microscopy. NLOM: nonlinear optical microscopy.

Taking advantage of most of these interactions, optical microscopy allows us to investigate changes at the molecular, cellular, and tissue levels. As shown in Figure 6, each of the most popular imaging modes employs vastly different illumination and detection strategies to form an image of different biological components. The figure illustrates biological tissue (represented by the ECM schematic) on a glass slide. The sample is being illuminated with total internal reflection (TIRFM), traditional widefield fluorescence microscopy (WFFM), laser scanning confocal microscopy (LSCM), and nonlinear optical microscopy (NLOM). The light penetration and regions where light interacts with the sample are indicated in red overlays.

In the next subsections, we present a description of each of the most common linear optical microscopy modalities, as well as examples of applications focused on imaging ECM components.

#### 2.1.1. Widefield Fluorescence Microscopy (WFFM)

The most basic form of fluorescence microscopy, widefield fluorescence microscopy (WFFM), bathes the entire sample in light, filtering excitation and emission light and observing through a microscope [45]. While the basic approach to WF fluorescence microscopy has not changed, the technology used in this method has advanced, improving imaging speed and quality [75]. However, while WFFM is simple and cheap to use, it has poor imaging depth and high signal-to-noise ratio [75].

WFFM has been used extensively in viewing the ECM. From viewing collagen fibril assembly [75] to viewing elastin fiber formation [58], WFFM has been a valuable tool in the cell biologist’s arsenal due to its simplicity and ability to excite and view fluorophores of the ECM. While WFFM is the simplest of the linear, diffraction-limited microscopy methods, it continues to be used to characterize and explore the ECM. For example, Xu et al. used chemometric WFFM to analyse thin sections of lung tissue, where they measured the relative concentrations of fluorophores over the relative intensities of emitted light [103]. Their improvements to WFFM improved the poor SNR usually found in WFFM. More recently, in 2020, WFFM has been used to characterize extracellular matrix scaffolds [104]. Riis et al. assessed the composition of extracellular matrix scaffolds produced by adipose-derived stem cells (ASC) [104]. Assessment of the scaffolds was done using WFFM to evaluate the amount of collagen and noncollagenous proteins found in the scaffolds. They found collagen I, collagen III, and fibronectin were well preserved after cellularization, forming networks that further supported the growth and proliferation of fibroblasts and endothelial cells, demonstrating ASC-derived ECM scaffolds as a viable biomaterial for further study.

#### 2.1.2. Total Internal Reflection (TIRF) Microscopy

TIRF Microscopy is a method very similar to that of WFFM but allows imaging of fluorophores very close to a surface while preventing imaging of fluorophores further from that surface [105]. Using a beam of excitation light oblique to the sample at an angle (the critical angle) to the coverslip ensures that light is internally reflected. This total internal reflection of light generates an electromagnetic field at the interface of the coverslip and sample, which then excites fluorophores [45]. Since this field is only generated very close to the coverslip, only fluorophores close to the interface are excited and fluoresce as such, TIRF microscopy is excellent for select imaging of cell-substrate interfaces, such as cell membranes [106].

Due to its narrow depth of excitation, TIRF microscopy is ideal for imaging cell-substrate interfaces, as mentioned previously. It has been used to map cell-substrate separation distances [107], showing the distance between cells and the basement membrane. In addition, TIRF microscopy has been used in the study of ECM degradation by invadopodia, protrusions of cellular plasma membranes found in cancer invasion and metastasis [59,108].

Vega et al. explored the effect of acetylation on the ECM kidney [90]. Using TIRF microscopy to image integrins, they were able to show that an increase in acetylation of integrins promoted an increase in fibronectin matrix assembly, building on their previous work demonstrating that glucose metabolism promoted fibronectin assembly through integrin activation. Thus, their work advanced the use of TIRF in the exploration of the cause of kidney diseases, especially diabetic nephropathy.

TIRF microscopy has also found use complementing other technologies, such as its use in guiding atomic force microscopy (AFM) in testing the biomechanical properties of the pericellular matrix of chondrocytes found in cartilages. Chery et al. used TIRF microscopy to explore the pericellular matrix (PCM), a narrow, specialized band of ECM found around chondrocytes of cartilage [109]. Using TIRF to image collagen IV, the resultant images were used to guide mechanical testing of the PCM using AFM, demonstrating the use of TIRF microscopy as a guide for other methods of ECM characterization.

In 2020, Umana-Diaz et al. demonstrated the use of time-lapse TIRF microscopy in exploring the role of Lysyl Oxidase-Like 2 (LOXL2), an enzyme responsible for the crosslinking of fibrillar ECM proteins, in the vascular basement membrane [110]. Using time-lapse TIRF microscopy, they demonstrated that LOXL2 was incorporated directly into the fibrillar structure and basal membranes of the ECM upon secretion by cells. They also showed that LOXL2 was found for extended periods of time post-secretion, suggesting it becomes bound to ECM proteins, such as collagen IV [110].

#### 2.1.3. Laser Scanning Confocal Microscopy (LSCM)

At its most basic form, confocal microscopy is very similar to widefield microscopy. The major difference between this technique and WFFM is that excitation light is passed through a pinhole aperture before reaching the sample, and a pinhole aperture is situated before the detector. This has the advantage of removing any emitted light that is out of focus [111]. As a result, fluorescence is only observed in the focal plane of the focused excitation light, producing a focused, narrow plane of imaging. This has several advantages over WFFM, where the entire sample is bathed in excitation light, and a significant amount of unwanted fluorescence is generated [111]. Since the excitation beam is narrow and targets a small region of the sample, it must be scanned across the sample to create an image. This is generally done with dichromatic mirrors that can move the excitation beam across the sample while allowing fluorescence to pass through to detection [111]. This scanning of the excitation beam gives us the name laser scanning confocal microscopy (LSCM). As imaging is restricted to a narrow plane, 3D images can be built by stacking the 2D images created by scanning, as moving the focal plane up and down through the image produces 2D images at different depths.

Due to its selectivity in only imaging the focal plane and relative ease of use, LSCM has found extensive use in imaging and characterization of the ECM. From the early use of LSCM in the exploration of the expression of fibronectins and laminins in the ECM, postimplantation of grafts in mice [91] to the characterization of artificial ECM constructs suitable for tissue engineering purposes [112] and in imaging perineuronal nets (~240), LSCM has found a wide range of application in cell and molecular biology and tissue engineering.

LSCM has continued to be a valuable tool in the characterization of ECMs, especially in vivo and in artificial matrix constructs. In 2021, Pínter et al. used LSCM in their study of proteoglycan distribution in the adult rat brain [78]. Using LSCM, they showed regional differences of chondroitin sulfate proteoglycan 5 (CSPG-5), potentially affecting the functioning of neural networks within the brain [107]. In 2020, Kaushik et al. used LSCM in their study of perineuronal nets found in neural ECM [5]. By imaging the PNN with LSCM, they were able to demonstrate morphological changes in the PNN of ketamine-treated rat schizophrenia models. Soria et al. explored the use of LSCM in their 2020 study of hyaluronic acid remodelling in neural ECM, demonstrating the interaction of the ECM and pathological states of the brain [82].

Hayes et al. also used LSCM in localizing the proteoglycan perlecan in intervertebral disks [79]. With LSCM, they were able to show perlecan surrounding chrondrons within the intervertebral disk, providing a potential new target for further study or treatments for intervertebral disk pathologies [79]. De Angelis et al. demonstrated the use of LSCM in imaging hyaluronic acid in their 2017 study of angiogenesis in zebrafish [83]. They showed that hyaluronic acid surrounds sprouting blood vessels, suggesting that hyaluronic acid has a significant role in angiogenesis. LSCM has also been used in exploring how inhomogeneities in 3D collagen matrices affect the mechanics of the matrix and how cancer cells migrate through these matrices [60]. LSCM was used in finding how gene silencing may affect the organization and regulation of ECM macromolecules, such as collagen and laminin [61]. LSCM has also found use in identifying ECM proteins, such as collagen, fibronectin, and laminin, in electrospun scaffolds [49] and in studying the effect of pluripotent stem-cell-derived beta cells on the ECM [62]. Overall, LSCM remains a viable and useful tool in the characterization of the ECM.

#### 2.1.4. Slit Scanning Confocal (SC) and Spinning Disk Confocal Microscopy (SDCM)

While LSCM is the most common form of confocal microscopy used today [113], other varieties of confocal microscopy have been used. Where LSCM uses a single beam to scan across a sample, multipoint confocal microscopy harnesses the use of multiple focal points to image samples. Spinning disk confocal microscopy (SDCM) achieves this with the use of a spinning disk with outwardly spiraling pinholes, as first developed by Nipkow in 1880 [113] and improved upon in the Yokogawa implementation. In the Yokogawa implementation, pinholes are placed in a spiral configuration, and to ensure that light is properly focused through the holes, they are complemented with a disk of microlenses [114]. This configuration of pinholes and microlenses ensures excitation light reaches the sample [114]. As with the emission pinhole found in LSCM systems, pinholes in the rotating disk reject out of focus light [114].

SDCM is faster than LSCM, making it suitable for imaging live cells [114] but can have issues with phototoxicity, visual artifacts, and crosstalk between pinholes [113]. An intermediary between LSCM and multipoint scanning microscopes is the slit scanning confocal microscope (SSCM). In this form of confocal microscopy, a slit aperture replaces the pinhole aperture of LSCM, and a narrow beam of light is scanned across the sample [115]. More of the sample is captured as the beam is scanned, giving the advantage of significantly increasing imaging speed but with lower resolution and rapid photobleaching of the sample [113].

In imaging live cells, Doyle et al. demonstrated the use of SDCM in their study of the migration of fibroblast and mesenchymal cells [63]. By imaging collagen using SDCM, they were able to show the migration of cells in 3D collagen constructs, proposing that mesenchymal cells generate forces on the ECM as they move through it. While SDCM is well suited to image live cells in vivo, SDCM has also found use in imaging macromoles of the ECM. Rácz et al. studied the organization of several biomolecules in the ECM of the red nucleus of rats [80]. Using SDCM, they explored the distribution of the proteoglycans aggrecan, versican, neurocan, and brevican and the distribution of hyaluronic acid within the ECM of the red nucleus. They demonstrated the use of SDCM in imaging proteoglycans, a group of biomolecules that are often overlooked in the characterization of the ECM.

#### 2.1.5. Structured Illumination Microscopy (SIM)

SIM is a super-resolution microscopy technique that uses a structured pattern of excitation light to illuminate the sample, usually a series of parallel lines, but other patterns may also be used [47]. As the illumination passes over the sample, moiré fringes are produced by both the overlying illumination and the fluorescence produced by fluorophores in the sample. As the patterned illumination is shifted and rotated, multiple images are captured, and high-resolution information encoded in these images is decoded by deconvolution [116]. Resolution improvements are approximately two-fold, resolving detail down to ~100 nm in the XY direction and ~400 nm in the Z direction [117].

SIM has found extensive use in imaging of cells and cellular processes due to its suitability in the imaging of live cells [118,119], imaging organelles such as the endoplasmic reticulum [120] and cytoskeleton [120,121]. In the characterization of cell-matrix interactions, SIM has been used in imaging integrins. Hu et al. used SIM in 2015 to discover that focal adhesions are made up of linear subunits [84]. As integrins have an important role in cell-matrix interactions, linking the outside of the cell to the inner, their characterization within focal adhesions is vital in understanding how these complexes mediate cell-matrix interactions. Hu et al. were able to demonstrate that focal adhesion were formed of several linear subunits after imaging with SIM [84].

As focal adhesions are relevant in the ability of a cell to respond to mechanical stress and generate mechanical stresses on the ECM, investigation of the structure of focal adhesions is critical in understanding how mechanical stresses and cells interact [84]. Dzyubenko et al. demonstrated the use of SIM in imaging the proteoglycan aggrecan and polysaccharide glycan in the perineuronal nets (PNNs) of the brain [81]. Perineuronal nets are a specialized form of highly structured and organised extracellular matrix found in neural tissues [81]. Using SIM, they were able to elucidate the structure of PNNs and demonstrated a depletion of PNNs following cerebral ischemia [81]. Their findings suggest that modification of PNNs following cerebral injury could support neural rewiring and return to function of brain tissues [81].

#### 2.1.6. Stimulated Emission Depletion Microscopy (STED) and Ground State Depletion Microscopy (GSD)

STED Microscopy is another super-resolution microscopy technique used to break the diffraction limit. It relies on the depletion of fluorophores in a torus around the focal point, leaving fluorophores at the focal point free to fluoresce upon excitation [122]. Using a torus of low-energy light around the focal point, fluorophores are excited but fail to reach an energy state that allows them to emit fluorescence [122]. At the same time, fluorophores within the focal point are excited with high-energy light, reaching an energy state that permits fluorescence [122]. This has the effect of localizing fluorescence only to the focal point, breaching the diffraction limit, and permitting the imaging of structures in the XY plane of approximately 40 nm in living cells and tissues [123] and approximately 20 nm in fixed samples [124]. A similar technique to STED is ground state depletion (GSD) microscopy. While STED switches off the ability for fluorophores to fluoresce, GSD forces fluorophores into a ‘triple state’, a long-lived dark state where fluorescence is unable to occur [125].

Much like other super-resolution methods, STED and GSD have been used extensively in the past and have found ongoing use in imaging live cells [126,127] and cellular components, such as actin in live cells [128], ribonucleic acids (RNA) in fixed cells [129], and the endoplasmic reticulum of neural cells [130]; they have found limited use in the characterization of the ECM.

STED microscopy has recently been used in the characterization of extracellular fluid flow in the extracellular space (ECS) of the brain. In 2018, Tønnesen et al. used 3D-STED to image diffusible fluorescent markers within the ECS, a method they termed ‘SUSHI’, producing clear images in a tissue that has defied conventional light microscopy in the past [50]. Using this method, they were able to glance into the complex world of the ECS and demonstrate the use of 3D-STED in characterizing the spatial dimensions of the ECS, both statically and dynamically. They proposed further uses of SUSHI in the characterization of other ECM elements of brain tissues, such as perineuronal nets, a specialized extracellular matrix [50], making SUSHI a powerful tool for future studies.

STED has also found use in imaging integrins, cell-surface receptors that directly communicate with extracellular components such as fibronectin. Spiess et al. demonstrated the use of STED in direct imaging of integrins, comparing images produced by STED microscopy to those produced by another super-resolution technique, STORM [85]. Their work demonstrated the use of STED in the characterization of cell–extracellular interaction sites structure and prompted further questions surrounding the nature of cell and ECM interaction.

#### 2.1.7. Photoactivated Localization Microscopy (PALM/fPALM)

PALM is a super-resolution technique able to target and image single molecules [131]. The fundamental method behind PALM is the summation of many different images over time. A small portion of fluorophores are excited, and their locations are imaged, upon which they photobleach [131]. Another set of fluorophores are then excited, imaged, and the image summed to the previous image [131]. Since captured fluorescence is diffraction-limited, the software is used to fit these spots to pinpoint locations [131]. This cycle is repeated, with subsequent images summed to produce a final image where all fluorophores are captured [131]. In this way, single fluorophores can be captured and imaged to precise locations [131]. Typically, PALM can resolve detail down to approximately within 10–15 nm [132].

PALM has found use predominantly in the imaging of the internal workings of the cellular environment. PALM has been used in imaging the proteins of organelles, such as mitochondrial proteins [133], cell-surface receptors [84,134], and cell junctions [135,136]. In imaging extracellular-related components, PALM has been used in imaging focal adhesions, targeting integrin, a protein found in the complex of a focal adhesion that interacts with extracellular components [137].

More recently, PALM has been used in further elucidation of the structure and function of integrins found in focal adhesions. Moore et al. used a variation on PALM known as iPALM, combining interferometry with PALM modalities [51]. Using iPALM, they were able to demonstrate that integrins undergo a conformational change upon interaction with the extracellular component fibronectin, showing that PALM is a viable technique to optically image changes in protein configuration [51]. In further exploration of how extracellular components and integrins interact, Changede et al. used PALM to image integrin cluster formation on different fiber formations [87]. Creating a substrate of parallel and crossing nanowires, they demonstrated that integrin clusters form where parallel wires were closely spaced or crossed [87]. Based on their findings, they suggested that an ECM with excessively deposited collagen/fibrosis or collagen fibers that are too large would affect the ability of cells to remodel the ECM [87].

#### 2.1.8. Stochastic Optical Reconstruction Microscopy (STORM/dSTORM)

Another similar method to PALM is stochastic optical reconstruction microscopy (STORM). Whereas PALM relies on the photobleaching of fluorophores, STORM relies on fluorophores that are reversibly switchable between on and off states [138]. Fluorophores are maintained in the dark state by the use of buffers, and a small subset switched on for imaging [138]. Imaging of the fluorophores is then done in the same process as PALM, with the summation of fitted images over time [138]. As with PALM, STORM can resolve down to approximately within 10–15 nm [132]. In 2008, Heilemann et al. developed a variation of STORM called direct STORM (dSTORM). The main difference is that dSTORM uses conventional photoswitchable fluorescent probes. Using these probes, one can have them reversibly cycled between a fluorescent and dark condition. It is achieved by using light with different wavelengths, and one of the main advantages is that it does not require an activator fluorophore [139].

As with PALM, STORM has considerable use in imaging cellular processes and structures. STORM has been used in imaging mitochondrial dynamics, targeting proteins within the mitochondria [140]. Imaging of the structure of DNA within the nucleus has also been achieved with STORM, targeting chromatin [141] or DNA directly [142]. STORM has also been used to map the 3D structure of microtubules in the cytoskeleton of epithelial cells [143].

Like PALM, STORM has been recently used in imaging integrins. Fan et al. used STORM to image integrins on the surface of neutrophils [88]. They demonstrated that integrins exist as a patterned array on the surface of neutrophils and showed that STORM is a viable technique in the exploration of molecule patterning in cell–matrix interactions [88]. Codron et al. used STORM to reveal the organisation of aggregates in the brain [92]. Aggregates in the brain are one of the hallmarks of Alzheimer’s disease, formed by extracellular deposits of Amyloid-β peptides (Aβ) and interneuronal aggregates of hyperphosphorylated tau protein (p.Tau) [92]. Codron et al. used STORM to explore the structure and organization of extracellular deposits by targeting Aβ and p.Tau, demonstrating the use of the super-fluorescent technique in observation of the structure of pathological structures within the brain [92].

STORM has also been used in the characterization of bone mineralization. Zhou et al. used STORM in their study of bone mineralization [93]. Using STORM, they were able to image the 3D distribution of polyaspartic acid and calcium around collagen fibrils. With these images and other images taken with transmission electron microscopes and force microscopy, they were able to demonstrate a viable process in mineralization of collagen scaffolds, showing that STORM can be used to target some more exotic fluorophores of the ECM [93]. Hydroxyapatite was the target for STORM imaging in another bone mineralization study carried out by Yao et al. in 2019 [94]. Studying the mineralization of collagen I fibrils in tendon, Yao et al. used 2D and 3D STORM to image the mineralization of the fibrils, showing that mineralization occurs on both the surface and inside of fibrils [94]. They also demonstrated the use of STORM in the characterization of an inorganic ECM fluorophore and its use in the characterization of the bone mineralization process. Another bone mineralization study was carried out by He et al. in 2020, using STORM to target and image chondroitin sulfate and its role in the intrafibrillar mineralization of collagen [52]. With STORM, they were able to prove that chondroitin sulfate acted as nucleation sites for intrafibrillar collagen mineralization and that it may be used to restore demineralized tissues [52].

#### 2.1.9. Fluorescence Lifetime Imaging Microscopy (FLIM)

FLIM is an imaging technique that relies on the difference in the decay rate of fluorescence [144]. FLIM is used to image the spatial distribution of fluorophore lifetimes. Fluorophores decay and release photons at different rates, most of the time within nanosecond ranges [144]. Since fluorophores decay at different times, the lifetime of the fluorophore can be used to map where fluorophores are found [144]. FLIM can be classified into frequency-based FLIM and time-domain FLIM [144]. In frequency-based FLIM, excitation light is pulsed, and the differences in fluorophore lifetimes produce emission signals with increasing phase difference and decreased intensity, in comparison to the excitation light [144]. The intensities and phase differences are then used to map the locations of fluorophores. In time-domain FLIM, fluorophores are excited, and the time and intensity of emission recorded and used to map the distribution of fluorophores [144]. FLIM initially used WFFM but has since expanded to use confocal microscopes, two-photon excitation, and multiphoton microscopes [145]. FLIM has a wide range of biomedical uses, offering quick detection speeds, and in the case of Multiphoton FLIM, offering the ability to image thick samples in vivo and in situ [146]. It has been used in imaging protein and protein interactions [147,148], in probing the intracellular processes of cancerous cells [149,150] and in the identification of the distributions of extracellular components, such as collagen [151] and elastin [152], or extracellular characteristics, such as pH [153]. 

FLIM has found recent use in imaging collagen and elastin of the ECM. In 2018 Li et al. used FLIM to study the extracellular matrix architecture of bovine pericardium under the effects of collagenase activity [64]. FLIM was used to determine collagen content of the extracellular matrix over time, as samples were digested over time by collagenases. They demonstrated the use of FLIM as an in situ, label-free, nondestructive technique of quantifying collagen distribution, positioning FLIM as a valuable tool in biomedical and tissue engineering applications [64]. Vazquez-Portalatin et al. used FLIM in their characterization of collagen and elastin blend hydrogels [65]. Autofluorescence of different hydrogels, with various ratios of collagen and elastin, was measured using FLIM, and changes in the autofluorescence lifetime suggested changes in the microenvironment of the hydrogels, relating it to a changing dynamic modulus.

Vazquez et al. demonstrated the use of FLIM as a nondestructive assessment of the mechanical properties of hydrogel [65]. Haudenschild et al. also demonstrated the use of FLIM in the characterization of matrix mechanical properties in their 2019 study of the effect of exogenous proteins in the production of self-assembled cartilage constructs [66]. They showed that the use of a combination of exogenous proteins LOXL2 and LINK produced robust cartilage constructs, with properties resembling those of cartilage found in vivo [66]. They also proved the use of fiber-optics-based FLIM instrumentation and developed machine-learning models to identify crosslinking in engineered cartilage, showing that FLIM-based tools could easily be used in manufacturing as a diagnostic tool and in future biomedical studies [66].

FLIM has also been used in imaging nonproteinous components of the ECM. In 2020, Okkelman et al. used FLIM to image the distribution of extracellular calcium ions, an important signalling ion [95]. The use of a biosensor enabled Okkelman et al. to visualize the distribution of extracellular calcium, showing a pool of extracellular calcium surrounding intestinal organoids. They demonstrated the use of FLIM in imaging biosensors that have a high potential for use in vivo, in organoid models, or for further study of tissue processes, such as healing [95]. FLIM was also used by Szmacinski et al. in their 2020 study of extracellular hydroxyapatite deposits in the retina [96]. A hallmark of age-related macular degeneration, hydroxyapatite deposits (known as drusen) are extracellular deposits found under the retina [96]. Using a tetracycline stain, Szmacinski et al. were able to demonstrate the use of FLIM in resolving the locations of hydroxyapatite deposits in the subretinal layer and its potential use as a tool in the diagnosis of other age-related eye diseases [96].

#### 2.1.10. Additional Modalities: Fluctuation-Based Super-Resolution Microscopy (FSM) and Pixel Reassignment Super-Resolution Microscopy (PRSM)

Fluctuation-based super-resolution microscopy (FSM) is a collection of techniques that rely on the small fluctuations in emitted light of fluorophores. Capturing these fluctuations over time, with tens to hundreds of images, and performing higher-order statistical analysis of those fluctuations produce images with improved resolution [154], and an enhancement of two- or three-fold can be expected [155]. Super-resolution optical fluctuation imaging (SOFI) is an FSM technique developed by Dertinger et al. used in several studies of focal adhesions [154]. SOFI was used by Deschout et al. as a complementary technique to PALM in imaging focal adhesions, targeting integrin β3, a subunit of integrins found in focal adhesions [89]. Combining SOFI and PALM provided an insight into the localization of focal adhesions, and Deschout et al. proved the use of SOFI as a viable technique in the localization of integrins and their role in cell–matrix interactions [89].

Pixel reassignment super-resolution microscopy uses one or multiple focal points to scan across the sample, much like confocal microscopy [47]. Fluorescence is then captured using an array detector [156] and detected pixels reassigned in space to increase resolution by 1.4-fold [157]. Several variants exist, such as image scanning microscopy, rescan confocal microscopy, and multifocal structured illumination microscopy, among others [47]. Pixel reassignment was used by Barlow et al. in 2020 to provide super-resolution measurements of collagen fibers [67]. In this study, they used an Airyscan detector, an array detector used with confocal microscopy [158], to provide the requisite array detector. Combined with multiphoton microscopy, they showed that pixel reassignment was able to image collagen fibers in the heart, lungs, and collagen gels with super-resolution. They then went on to show that pixel-reassignment super-resolution images could be used to improve directionality metrics of collagen fibers, and super-resolution images of elastin were also easily obtainable. This demonstrated the case and viability of pixel reassignment in the characterization of extracellular biomolecules, especially collagen and elastin [67].

### 2.2. Nonlinear Optical Microscopy (NLOM)

NLOM, also known as multiphoton microscopy, uses nonlinear interactions between light and matter to generate images in contrast with conventional microscopy, which typically uses linear interactions (absorption, scattering, refraction, and fluorescence). NLOM is a group of techniques that rely on nonlinear optical effects to image proteins, cells, and other endogenous and exogenous fluorophores.

In imaging and characterization of the ECM, NLOM has found significant use. NLOM has been used in the imaging of the ECM of embryoid bodies, exocrine tissue, cartilage, and heart valves [159]. NLOM has been used in exploring how cancer invasion and proliferation may affect change on the ECM [160,161] and in the evaluation of medical conditions [162,163,164]. NLOM has continued to find use in the characterization of the ECM, with application in the imaging of fibrillar collagens, elastin fibers, fibronectin, and other fibrillar proteins [39,41,165]. NLOM has recently been used in the evaluation of the ECM of an aging aorta, and we often find the combination of multiple modalities such as two-photon excitation fluorescence (TPEF) and second-harmonic generation (SHG) to assess changes related to elastin and fibrillar collagen in biological tissue.

Using NLOM, Cavinto et al. (2021) were able to explore how the ECM changes over time, targeting the collagen and elastin fibres of the murine aorta, to understand how aging affects the aorta and how biaxial loading changes the microstructure of the ECM [68]. They demonstrated that the ratio of collagen and elastin changes over time in different regions of the aorta, collagen fiber bundles thickened and straightened, while elastic lamellae appeared not to change. Jadidi et al. (2021) also explored the use of NLOM in characterization of elastin fibers in the ECM of femoral arteries, exploring how aging affects arterial stiffness and how the ECM changes with time [69]. They characterized how the ECM changes and remodels over time, with a hope that they could use their findings to further inform arterial models, especially in arteries that are prone to atherosclerosis. NLOM has found use in predicting the risk of breast cancer, as Xi et al. found in their exploration of how certain collagen configurations may affect cancer invasion [70]. They found five ‘collagen signatures’ associated with the invasion front of breast cancer cells and, by using NLOM images, were able to train a model to provide a signature score, giving a personalized approach to the prognosis and treatment of breast cancers. Similarly, Gubarkova et al. used NLOM to characterize elastin fiber morphology in different forms of breast cancer, demonstrating that NLOM can be used in the morphological characterization of different states of the same disease [76].

#### 2.2.1. Two-Photon Excitation Fluorescence (TPEF)

One of the most well known nonlinear optical microscopy modalities is two-photon excitation fluorescence (TPEF), a technique used in life science for several decades after Denk et al. first demonstrated the use of two-photon excitation in living cells [53]. Since then, TPEF has been a valuable tool in the life scientist’s toolbox.

The nonlinear effect upon which TPEF relies is similar to one-photon excitation fluorescence (OPEF), found in conventional fluorescence microscopy. While one photon is absorbed and emitted in OPEF, more than one photon is absorbed in TPEF. While only one photon is emitted, multiple photons may be absorbed by the fluorophore. As multiple photons are absorbed, lower intensities of light may be used in the excitation of fluorescence [166]. TPEF offers several advantages over OPEF. It has a high penetration depth, and as fluorescence is limited to a small area in the focal plane, no out-of-focus signal is generated, and the need for pinholes to block background light is removed [166]. TPEF also frequently takes advantage of endogenous fluorophores, limiting the need for exogenous fluorescent chemicals [166]. It has 3D scanning capabilities and is easily applied in a wide range of sample types [166]. However, there is a risk of photodamage at the focal point [166].

TPEF continues to be a powerful tool in the quantification of the ECM and has found several recent uses in mapping the ECM. In 2021, Benbouja and Hartnick used TPEF to quantify the morphology of the ECM in human vocal cords [77]. TPEF was used alongside other nonlinear optical methods to characterize the structure and organization of the ECM of the vocal folds, especially in targeting the elastic fibers found in the ECM. They found that the ECM of human vocal folds has several distinct regions, which may explain why and where some pathologies occur and demonstrated the use of TPEF in mapping the architecture of the vocal fold in real-time during surgeries [77]. Hsaio et al. used TPEF in their improved evaluation of liver fibrosis [71]. TPEF was used to image the structure of fibrotic liver tissue, and by using an auto-correction algorithm they developed, the accuracy and efficiency of quantification of liver fibrosis improved. They demonstrated that quantification of the state of tissue can be performed using TPEF and can be improved using post imaging algorithms [71].

#### 2.2.2. Multiharmonic Imaging Microscopy (MHIM)

While TPEF relies on the absorption of two or more photons to change the energy state of a fluorophore to an excited state, multiharmonic generation (MHG) relies on the absorption of two or more photons to bring the fluorophore to a virtual state [167]. The photon released when the fluorophore relaxes back to a ground state has double (or triple) the frequency of the photon that was absorbed, giving MHG its alternative name—frequency doubling (or tripling). MHG, as a nonlinear phenomenon, is used in imaging, known as multiharmonic imaging microscopy (MHIM) and has found great use in biological imaging. MHIM techniques can be split into two groups, each using a different form of harmonic generation [167].

Second harmonic generation (SHG) relies on the doubling of the frequency of excitation photons and finds particular use in the imaging of noncentrosymmetric structures, such as fibrillar collagen [168]. SHG has several advantages, such as low risk of phototoxicity, ability to be used in vivo, in vitro, and in fixed samples, and being able to detect the orientation of fibers but suffering from low signal intensity [169]. Third harmonic generation (THG) produces fluorescence photons with triple the frequency of excitation photons and finds particular use in the detection of inhomogeneities in tissue, such as the interface between a lipid body and the surrounding media [170]. THG has several advantages, many of which are similar to SHG, with the addition that THG is completely label-free. However, emitted photons have fairly large wavelengths in the far-infrared region [169].

SHG has found considerable use in imaging of fibrillar collagen found in the ECM since Campagnola et al. demonstrated the use of SHG in the imaging of fibrillar collagen, acto-myosin, and tubulin [41] in biological tissues. When combined with other NLOM modalities, SHG was found to be a suitable method for imaging the ECM in living cells in collagen gel models [171]. SHG imaging has found considerable use in imaging ECM alteration caused by cancer invasion [172,173], in ECM remodelling caused by fibrosis [174,175], and in the exploration of the role of the ECM in wound repair [176,177]. THG has been used predominantly in imaging cell membranes [178,179], lipid bodies [170,180], and mineralized structures, such as enamel [181].

In 2020, Atkuru et al. explored the effect of cellular aging on oral extracellular matrix organisation [72]. Using SHG imaging, they evaluated the effects of aging on oral fibroblasts’ ability to produce and organize fibrillar collagen in culture. When combined with other fluorescence techniques to access other ECM proteins, they found that cellular aging has a detrimental effect on ECM production and organization in oral tissues and may provide a basis for future treatments targeted at improving outcomes for elderly patients.

The role of collagen fiber morphology on the migration of ovarian cancer cells was explored by Alkmin et al. Using SHG imaging, they analysed and characterized the morphology of collagen fibers in normal ovarian tissues, high-risk tissues, and benign and high-grade tumors [55]. Once imaged and characterized, these collagen morphologies were used as blueprints in the construction of ovarian tissue models, and the effect of morphology on cancer cell migration was studied. Alkmin et al. demonstrated the use of SHG imaging to produce high-resolution images, that when combined with other methods, could be used to produce accurate tissue models.

Finally, a variant of SHG imaging, known as polarization-dependent SHG imaging has recently been used in the characterization of ECM. Polarization-dependent SHG uses polarized excitation light and can be used to detect the orientation of collagen fibrils [182]. Pendleton et al. demonstrated the use of polarization-dependent SHG imaging as they explored how collagen fibers organized at different angles were remodelled into sheet structures [73]. They were able to find clear regions where remodelling of the bone ECM had produced those sheet structures of mineralized collagen fibers, suggesting that polarization-dependent SHG imaging could be used to evaluate bone health. Polarization-dependent SHG has even more recently been used in the three-dimensional evaluation and characterization of cell-seeded collagen scaffolds by Xydias et al. [74]. Using polarization-dependent SHG imaging, they were able to characterize changes in the collagen fiber structure of cell-seeded collagen-glycoprotein constructs over ten days. While this is a fairly standard use of SHG image, they extended this to characterization of the 3D structure of collagen fibers within the constructs and demonstrated clear remodelling of collagen within the constructs. Xydias et al. showed that polarization-dependent SHG imaging is a powerful tool in the characterization of the ECM, especially in imaging fibrillar collagen.

### 2.3. Raman-Based Modalities

Coherent anti-Stokes Raman scattering (CARS) as a physical phenomenon has been harnessed as an imaging modality since the first CARS microscope was constructed in 1982 [183]. This modality relies on the phenomena of scattering, produced when a photon interacts with a molecule, organic or inorganic, to change its vibrational state [183]. When the molecule relaxes back to a ground state, a photon is released, making the process similar to fluorescence but without changing the electronic state of the molecule [100]. While the scattered photon can have the same frequency as the photon that interacts, Raman scattering produces photons with higher (Stokes Raman) or lower (anti-Stokes Raman) wavelengths [100]. This is harnessed in CARS microscopy, using three lasers to elicit scattered light with a lower wavelength, with the same frequency and same difference in phase, known as coherence [100]. CARS microscopy is very effective in imaging lipids and is usually used to image cell membranes, lipid droplets, and other lipid-based structures [100]. Like other NLOM, CARS microscopy provides high resolution and penetration in thick samples, minimizes photobleaching and phototoxicity to allow for long-term imaging, and is label-free [184]. However, CARS microscopes are limited in their use due to their expense and have weak signals in comparison to fluorescence [184].

CARS microscopy is commonly used as a label-free, noninvasive technique for imaging live cells and internal cellular processes, as it is well suited to generating and receiving signals from the phospholipids that surround cells and cell organelles [185,186,187]. CARS microscopy is frequently used as a complementary imaging modality to other NLOM modalities, allowing the localization and morphology of cells when studying the structure of the ECM [188,189]. CARS microscopy can also be used to image extracellular lipids, such as lipid crystals [190], and other extracellular components, such as elastin [191].

Recent uses of CARS microscopy have targeted a wider range of biomolecules than lipids, such as GAG chains, commonly found to be attached to proteins in proteoglycans. Lima et al. explored the use of GAG probes for use in CARS microscopy [97]. Usually unable to be selectively targeted by imaging modalities, Lima et al. developed probes based on fluorine-19 and used them to selectively image GAGs chains, proving the use of CARS microscopy in imaging biomolecules that usually cannot be selectively imaged [97]. More recently, Sehm et al. demonstrated the use of CARS in imaging extracellular lipid deposits and cholesterol crystals in the walls of blood vessels following an aneurysm [98]. By harnessing the ability of CARS microscopy in imaging lipids, they were able to show extracellular lipid and cholesterol crystal deposition in a vessel aneurysm, along with other multiphoton microscopy techniques, to demonstrate changes in the microstructure of the vessel walls in an aneurysm [98]. As part of a suite of multiphoton modalities, CARS was shown to be an excellent contributor in label-free imaging and characterization of morphological and microstructural changes in vessel pathologies.

## 3. Conclusions

The ECM plays an important role in regulating tissue and cellular functions in the body. ECM homeostasis is essential for wound healing, development, and normal organ functioning. Different chronic and inflammatory diseases such as asthma, chronic obstructive pulmonary disease (COPD), cystic fibrosis, and even some cancers present significant changes in the ECM structure. Therefore, having specific tools to image the ECM components as well as cells specific to tissues is essential. Optical microscopy provides insights into the spatial, temporal, and molecular changes happening in the ECM as well as regarding the cellular adhesion points, cytoskeleton morphology, and other features of such a complex microenvironment. Recent developments and applications of optical imaging have been used to address increasingly complex biological questions related to ECM remodelling, and a collection of representative studies was presented in this review. We discussed the main advantages and limitations of the mains optical microscopy modalities and provided an overview on how these techniques can support advances in biology by allowing us to visualize structural changes and mechanisms crucial to tissue remodelling. While imaging techniques and technologies improve, image analysis algorithms represent another area of continued interest. From deep-learning models to neural networks, improvements in ECM image analysis furthers our ability to explore and characterize the fundamental structures of tissues and organs [192,193]. The development of open-source tools and plugins to accurately track structural changes of the ECM are welcome and needed as we attempt to establish patterns regarding the analysis of such features [38,194,195]. Now, more than ever, software and hardware development are advancing hand in hand. The development of more powerful microscopy techniques is largely benefiting from the application of robust algorithms based on machine learning and other strategies, which are allowing us to obtain features and quantify subtle changes. This combination of powerful imaging modalities with innovative analysis approaches as well as the development of probes and extrinsic labels is allowing us to understand ECM remodelling mechanisms in health and pathological conditions, with a level of detail not available before.

## Figures and Tables

**Table 1:** The most commonly used linear fluorescence-based and diffraction-limited microscopy modalities to assess ECM components.

Technique	Main Advantages	Main Limitations	ECM and Non-ECM Components Commonly Imaged
WFFM	Possible to observe samples directly in the microscope ocular Low maintenance cost	Significant background interference Risk of channel-to-channel bleed-through Excitation wavelength bands depend on filter sets available	Collagen Elastin Cytoskeleton (actin) Cell adhesion points
TIRFM	Good SNR due to low penetration depth of the evanescent field Reduced photobleaching	Sectioning is confined to a single-plane immediately adjacent to the cover glass or slide	Fibronectin Crosslinking of ECM fibrillar proteins Cell adhesion points Endo-exocytosis Cytoskeleton dynamics
LSCM	Superior image quality when compared to WFFM No significant image blurring More flexible excitation/emission wavelengths (reduced channel-to-channel bleed-through) Magnification can be adjusted electronically Optical sectioning in the *z*-axis 3D reconstruction possible Suitable to image live cells Can be combined with SIM	Time-consuming (depending on the scanning speed) Limited number of excitation wavelengths available with common lasers Higher overall maintenance cost	Fibronectins Laminins Collagens Elastin Cytoskeleton proteins Proteoglycans Hyaluronan
SC/SDCM	Larger field of view. Significantly increased imaging speed. Suitable to image live cells.	Rapid photobleaching. Lower sectioning resolution along the z-axis when compared to LSCM. Multicolor applications are complex (need for multiple cameras, dichroic mirrors, and filter wheels).	Collagens Fibronectin Laminins Proteoglycans Hyaluronan

SNR: signal-to-noise ratio; WFFM: widefield fluorescence microscopy; TIRF: total internal reflection fluorescence microscopy; LSCM: laser scanning confocal microscopy; SC/SDCM: slit confocal/spinning disk confocal microscopy.

**Table 2:** The most commonly used linear fluorescence-based super-resolution microscopy modalities to assess ECM components.

Technique	Main Advantages	Main Limitations	ECM and Non-ECM Components Commonly Imaged
SIM	Improved resolution (~100 and 250 nm in the lateral and axial directions) Fast imaging rate Conventional fluorophores can be used Possible use of simultaneous fluorophores	Reduced resolution compared to other super-resolution modalities Vibration must be considered Imaging artifacts due to image processing Sensitive to out-of-focus light Longer processing times when compared to other modalities	Focal adhesions (integrins) Proteoglycans
STED	Confocal-based: fast scanning over small regions No need for specialized fluorophores Laser power tunes resolution Suitable for in vivo applications, where higher temporal resolution is needed No need to computationally reconstruct images	Very high laser intensities required for highest resolutions Vibration must be considered Photobleaching and phototoxicity must be considered Slight improvement in z-resolution when compared to LSCM	Cell-matrix interactions Extracellular fluid flow in the extracellular space Focal adhesions (integrins) Cell-surface receptors Fibronectin
STORM	High spatial resolution (~20–50 nm) Single fluorophores are imaged (important for quantitative imaging) Simple control of fluorophores (not limited to those that can undergo photoswitching, photoconversion, or photoactivation) Lower laser intensity can be used compared to STED (more suitable for in vivo applications)	Low temporal resolution Vibration must be considered Special fluorophores required Phototoxicity associated with multiple imaging/quenching cycles Imaging of regions close to the coverslip Extensive post-acquisition image processing is required Samples can be prone to drift (this can be corrected in most cases)	Actin cytoskeleton Cell-matrix interactions Inorganic ECM fluorophores (bone mineralization) Cell junctions
PALM	High spatial resolution (~20 to 50 nm) Single fluorophores are imaged (important for quantitative imaging) Simplicity in both concept and instrumentation, requiring only a modified widefield fluorescence microscope (to conduct single-molecule imaging) Ability to express fluorescent fusions in adherent cell cultures	Low temporal resolution Vibration must be considered Special fluorophores required Extensive post-acquisition image processing is required Samples can be prone to drift (this can be corrected in most cases)	Focal adhesions (integrins) Cell-surface receptors Cell junctions Mitochondrial proteins
FLIM	Ability to detect changes in the molecular environments of fluorophores Provide information about fluorophore function and behavior (not possible with intensity measurements alone) Do not require the throughput calibration steps that are needed for intensity-based experiments Provides better SNR for weakly fluorescent samples Estimates multiple lifetime components Minimizes the effect of photon scattering in thick layers of sample	Requires costly pulsed lasers Poor performance with high photon count rates or dynamic samples Localized environmental factors, such as autofluorescence or a change in pH, can also shorten the measured fluorescence lifetime and lead to artifacts	Collagens Elastin Extracellular calcium ions Hydroxyapatite deposits

SNR: signal-to-noise ratio; SIM: structured illumination microscopy; STED: stimulated emission depletion; PALM: photoactivated localization microscopy; STORM: stochastic optical reconstruction microscopy; FLIM: fluorescence lifetime imaging microscopy.

**Table 3:** The most commonly used nonlinear optical microscopy (NLOM)* modalities to assess ECM components.

Technique	Main Advantages	Main Limitations	ECM and Non-ECM Components Commonly Imaged
TPEF	Narrow localization of focal point Absence of out-of-focus absorption Frequently takes advantage of endogenous contrast mechanisms Effects of light scattering are less detrimental to two-photon microscopy than to LSCM Reduced phototoxicity Increased tissue imaging depth Ability to initiate localized photochemistry	Limited capability to image thick samples Increased photobleaching and photodamage in the focal plane when compared to single-photon fluorescence Evidence shows influence of pulse duration (as the pulse width becomes smaller the rate of cell damage) Heating, especially in pigmented samples, such as haemoglobin, melanin or chlorophyll	Elastin Intrinsic fluorophores (e.g., NADH, Flavin, and aromatic amino acids) Exogenous fluorophores (e.g., fluorescein, calcein, and quantum dots)
SHG (SFG)	It does not present significant phototoxicity or photobleaching relative to fluorescence methods Exogenous molecular probes are not required High-resolution imaging to depths of several hundred microns	Limited applicability to a small number of structural proteins Limited capability to image thick samples	Fibrillar collagens DNA Myosin Polysaccharides Microtubules
THG (SFG)	Exogenous molecular probes are not required Good SNR in isotropic scattering media	In scattering media, temporal pulse broadening is significant	Interfaces and optical inhomogeneities Cell organelles Red or white blood cells Lipid droplets Adipose tissue Axon myelin sheath Bone
CARS	Can stimulate the production of a significantly larger amount of signal than spontaneous Raman microscopy Does not require the introduction of exogenous dyes or markers High spatial resolution Fast signal detection	Presence of coherent nonresonant background Samples must be illuminated within certain phase-matching conditions Costly and difficult implementation when compared to linear techniques, fluorescence or SHG microscopy	Chemical information of some proteins and lipid-rich structures (e.g., myelin, cell membrane, and foam cells)

ECM: extracellular matrix. TPEF: two-photon excited fluorescence. SFG: sum-frequency generation. SHG: second-harmonic generation. THG: third-harmonic generation. CARS: coherent anti-Stokes Raman scattering. LSCM: laser scanning confocal microscopy. NADH: nicotinamide adenine dinucleotide + hydrogen. *NLOM is commonly also referred to as multiphoton microscopy.

**Table 4:** Representative examples of applications of different microscopy modalities to assess ECM main components.

ECM Component	Imaging Modality	Representative References
Collagen	WFFM, LCSM, SHG	[49,55,58,59,60,61,62,63,64,65,66,67,68,69,70,71,72,73,74]
Elastin	WFFM, LCSM, TPEF	[68,69,70,71,75,76,77]
Fibronectin	LCSM	[49,58,62,72]
Laminins	LCSM	[49,61,62,72]
Proteoglycans	LCSM, SIM, CARS, DSCM	[74,78,79,80,81]
Hyaluronan	LSCM, DSCM,	[80,82,83]
Cell-ECM interactions	STED, PALM	[50,84,85,86,87,88,89]
Other EC components	PALM, STORM, TIRF, CARS, THG	[52,90,91,92,93,94,95,96,97,98]

ECM: extracellular matrix; EC: extracellular; WFFM: widefield fluorescence microscopy; TIRF: total internal reflection microscopy; LSCM: laser scanning confocal microscopy; SDCM: spinning disk confocal microscopy; STED: stimulated emission depletion; PALM: photoactivated localization microscopy; STORM: stochastic optical reconstruction microscopy; TPEF: two-photon excited fluorescence. SHG: second-harmonic generation; THG: third-harmonic generation; CARS: coherent anti-Stokes Raman scattering.
